# Multidisciplinary Management of a Pregnant Trauma Patient With Gestational Diabetes: A Case Report on Combined Regional Anesthesia

**DOI:** 10.7759/cureus.68914

**Published:** 2024-09-07

**Authors:** Khaled AlManea, Chadi Abouras, Abdulrahman AlJamous, Maram Alnemer, Khalid Alqahtani

**Affiliations:** 1 Anesthesia, King Abdulaziz Medical City Riyadh, Riyadh, SAU; 2 Anesthesia and Critical Care, King Abdulaziz Medical City Riyadh, Riyadh, SAU; 3 Nursing, King Abdulaziz Medical City Riyadh, Riyadh, SAU

**Keywords:** multiple trauma, perioperative pain management, post anesthesia care unit (pacu), pregnant for non-obstetric surgery, regional anesthesiology

## Abstract

This clinical case report describes the management of a 36-year-old pregnant female at 36 weeks gestation, who was admitted to King Abdulaziz Medical City following a motor vehicle accident. The patient, with a history of gestational diabetes mellitus, sustained multiple fractures requiring surgical intervention. A combined spinal and supraclavicular block was chosen for anesthesia, with a contingency plan for general anesthesia and emergency cesarean section if needed. The surgical procedures were completed successfully, and the patient was stable postoperatively.

## Introduction

Trauma during pregnancy is a significant cause of maternal and fetal morbidity and mortality, particularly in the third trimester when physiological changes complicate clinical management [1,2]. Anesthesia in such cases must account for maternal safety while minimizing fetal risk. This report highlights the anesthetic approach and surgical management of a pregnant woman with gestational diabetes mellitus who sustained multiple traumatic injuries [3].

## Case presentation

A 36-year-old female, at 36 weeks gestation, presented to King Abdulaziz Medical City after a motor vehicle accident in the E-FAST (Extended Focused Assessment with Sonography in Trauma), there was absent lung sliding in the right chest, otherwise negative. The patient, known to have gestational diabetes mellitus, was diagnosed at 30 weeks of gestational age, she was on metformin 500 mg once daily oral route, plus diet, then since she was admitted to the hospital after the accident she was shifted to insulin infusion as per the hospital protocol. She has sustained multiple fractures, including rib, radius, and tibia fractures.

The fracture on the rib was a nondisplaced fracture of the left anterior seventh rib, no need for surgical intervention as per the orthopedic team without flail segment. The fracture on the left proximal radius with evidence of intra-articular extension was associated with volar displacement and angulation. The fracture of the tibia was a comminuted depressed lateral right tibial plateau fracture in keeping with the type II Schatzker classification.

Her weight was 95 kg, and she was managed with a diabetic protocol (D5 1/2 NS 1L + KCL 15 mEq with side drip of NS 100 ml + regular insulin 100 units at 0.5 unit/h) and a cervical collar. The obstetrics and gynecology team was consulted for fetal assessment and maternal clearance for surgery (Table 1) [1,4].

**Table 1:** Patient Demographics and Clinical History

Parameter	Details
Age	36 years
Gestational age	36 weeks
Weight	95 kg
Medical history	Gestational diabetes mellitus
Fractures sustained	Rib, radius, tibia

The anesthesia strategy included the use of both spinal and supraclavicular blocks. The patient was briefed on the associated risks, which included the potential need to switch to general anesthesia if spinal anesthesia not working and the possibility of requiring an emergency cesarean section. Following the patient’s informed consent, the operating room was meticulously prepared with all necessary emergency medications and monitoring equipment on hand.

Spinal anesthesia was administered at the L3-L4 level with 2.2 mL of 0.5% bupivacaine and 15 mcg fentanyl. The patient remained stable throughout the 3-hour procedure for tibia fracture fixation. Following this, a supraclavicular block using 20 mL of 0.75% ropivacaine was performed under ultrasound guidance. The block was effective on the first attempt, and the patient remained stable during the 2-hour surgery for the rib and wrist fractures. The total operative time was 5 hours and 22 minutes. Blood glucose was monitored hourly, with the final level recorded at 5.1 mmol/L (Table 2).

**Table 2:** Intraoperative and Postoperative Monitoring

Parameter	Baseline	Intraoperative	Postoperative
Blood glucose (mmol/L)	5.5	4.8-5.1	5.1
Blood pressure (mmHg)	120/80	110/70 – 130/85	120/80
Heart rate (bpm)	85	80-90	88

Postoperatively, the patient was transferred to the post-anesthesia care unit (PACU), where she remained stable and pain-free before being moved to the ward [5].

## Discussion

Managing trauma in pregnancy requires a multidisciplinary approach to ensure both maternal and fetal safety [1,6]. Regional anesthesia is often preferred over general anesthesia in such cases, as it reduces fetal drug exposure and allows for continued maternal and fetal monitoring. Spinal anesthesia was chosen for this patient due to its rapid onset and reliability in providing adequate surgical anesthesia, while the supraclavicular block provided effective analgesia for upper extremity surgery [5,3]. The patient’s obesity and gestational diabetes were additional factors influencing anesthesia management, requiring careful monitoring of blood glucose and medication dosing [1]. Continuous fetal monitoring, collaboration with the obstetrics team, and readiness for emergency interventions were critical to the positive outcome observed in this case.

The management of trauma in pregnant patients, particularly in the third trimester, presents unique challenges that require a careful balance between maternal and fetal safety. In the presented case, the combined use of spinal and supraclavicular blocks demonstrates a tailored approach to regional anesthesia, which aligns with the recommendations in the existing literature emphasizing the importance of minimizing fetal drug exposure and allowing for continuous monitoring of both mother and fetus.

Regional anesthesia is preferred in such cases because it reduces the potential risks associated with general anesthesia, such as airway complications and the potential for difficult intubation, which are exacerbated by the physiological changes of pregnancy [1]. In this case, the decision to use spinal anesthesia for the lower extremity procedure and a supraclavicular block for upper extremity surgery is consistent with the recommendations for using region-specific anesthesia to minimize the dose of anesthetics and provide effective analgesia [5].

The literature also emphasizes the need for a multidisciplinary approach in managing pregnant trauma patients, particularly when comorbidities like gestational diabetes are present. Gestational diabetes adds complexity to anesthetic management due to the need for stringent glucose control to prevent hyperglycemia or hypoglycemia, which could adversely affect both maternal and fetal outcomes. The case report’s management strategy of using a diabetic protocol and regular glucose monitoring aligns with standard practices discussed in the literature, ensuring optimal intraoperative glycemic control.

Furthermore, continuous fetal monitoring is a critical aspect of managing trauma in pregnancy, which includes the importance of close collaboration between the anesthesia and obstetrics teams to promptly address any signs of fetal distress [4]. The successful outcome in this case, with the patient remaining stable throughout the procedure, reflects the effectiveness of this multidisciplinary approach.

In comparison with similar cases in the literature, the anesthetic and surgical management detailed in this report exemplifies current best practices, highlighting the necessity of individualized care plans that consider both maternal and fetal needs. This case contributes to the growing body of evidence supporting the use of regional anesthesia in pregnant trauma patients, particularly when complicated by conditions like obesity and gestational diabetes.

## Conclusions

This case underscores the importance of a multidisciplinary and individualized approach to managing pregnant trauma patients. The use of combined regional anesthesia techniques, careful intraoperative monitoring, and collaboration among specialties ensured a successful outcome for both mother and fetus.
